# Assessment of diabetic distress and disease related factors in patients with type 2 diabetes in Isfahan: A way to tailor an effective intervention planning in Isfahan-Iran

**DOI:** 10.1186/2251-6581-11-20

**Published:** 2012-10-17

**Authors:** Azar Tol, Abdolvahab Baghbanian, Golamreza Sharifirad, Davoud Shojaeizadeh, Ahmadali Eslami, Fatemeh Alhani, Mohamadreza Mohajeri Tehrani

**Affiliations:** 1 Department of Health Education and Promotion, School of Public Health, Isfahan University of Medical Sciences, Ground Floor, School of Public Health, Hezarjarib Ave., P.O. Box: 8174673461, Isfahan, Iran; 2 Health Promotion Research Center, Zahedan University of Medical Sciences, Zahedan, Iran; 3 School of Public Health, Tehran University of Medical Sciences, 4th Floor, School of Public Health, Pour Sina Ave., Tehran, P.O. Box: 1417613191, Iran; 4 School of Medicine, Tarbiat Modarres University, Jalal-All-Ahmad, Tehran, P.O. Box: 14115331, Iran; 5 Endocrine and Metabolism Research Centre, Tehran University of Medical Sciences, 5th floor, Dr. Shariati Hospital, Northen Karegar Ave., P.O. Box: 1411413137, Tehran, Iran

**Keywords:** Diabetes distress, Emotional burden, Interpersonal distress, Physician distress, Regimen distress, Type 2 diabetes

## Abstract

**Background:**

The purpose of this study was to assess diabetes distress and its related factors among type 2 diabetic patients to better tailor intervention planning in Isfahan-Iran.

**Methods:**

A cross-sectional study was conducted in 2011. Study population was patients with type 2 diabetes referring to Omolbanin, an outpatient diabetic center in Isfahan. 140 diabetic patients met the inclusion criteria and were all included in the study. Patient’s diabetes distress was measured by DDS. A 17-item self-report diabetes distress scale was used with subscales reflecting 5 domains: 1) Emotional burden (5 items), 2) Physician distress (4 items), 3) Regimen distress (5 items) and 4) Interpersonal distress (3 items). The responses to each item were rated between 1 and 6 (1 = not a problem, 2 = a slight problem, 3 = a moderate problem, 4 = somewhat serious problem, 5 = a serious problem, 6 = a very serious problem). The minimum and the maximum of score in the scale were 17 and 114 respectively. Collected data was analyzed by using SPSS software version 11.5.

**Results:**

Mean age of participants were 53.23 years (SD = 7.82). 54.3% was female, 97.1% was married, and 57.1% had education lower than diploma. The average score of total diabetes distress was 2.96 ± 0.83. The average score of each domain was (3.40 ± 1.18), (2.57 ± 0.88), (2.97 ± 0.90), (2.76 ± 0.91) respectively. ‘Emotional Burden’ was considered as the most important domain in measuring diabetes distress. Total diabetes distress had significant association with age (p = 0.02), duration of diabetes (p<0.001), marital status, comorbidity, complications (p<0.001), and history of diabetes (p = 0.01). Pearson correlation coefficient revealed that diabetes distress of type 2 diabetic patients has a linear and direct relation with HbA_l_c (r = 0.63, p<0.001).

**Conclusion:**

It seems some keywords have a main role in diabetes distress such as emotional support, communication with patient and physician, self-efficacy and social support. All of these points are achievable through empowerment approach in diabetes care plan.

## Introduction

Type 2 diabetes is one of the most serious health concerns worldwide [1]. It is estimated that a 54% increase will occur in the number of adult patients living with diabetes from 2010 to 2030 [2]. Diabetes poses a big burden on individuals, families and societies [3]. Living with diabetes poses significant influence on people suffering from the disease. In the face of this situation, particularly when it comes to self-care practices, patients may become disturbed, upset, or depressed [4]. Diabetic patients may also suffer from diabetes-related distress – a condition where patients are concerned with the management of their diseases, getting the support they need, managing the emotional burden of diabetes, as well as access to needed care, conditions that are distinct from depression [5].

Healthcare professionals and researchers have identified many relevant factors that cause diabetes distress; the diagnosis, signs and symptoms of the diseases, to mention only some [6]. Diabetes-related emotional distress ranges from limited psychological problems to constant diabetes-related self-care behaviors such as regular blood sugar control, medications administration, insulin injection, and adherence to treatment regime [7]. Many studies have revealed that distress can significantly affect diabetic patients’ health outcomes, especially their self-management [8]. Findings of a qualitative study revealed that three subjects are closely related to diabetes distress including: 1) behavior pressure, 2) emotional pressure and 3) fear of diabetes complications [9].

In addition, findings from a complete survey which was performed in 13 countries showed that psychological issues such as diabetes distress are very prevalent among diabetic patients and can significantly affect the self-care function of the diabetic patients [10]. Diabetes distress poses additional constraints on patients and health care systems. Healthcare professionals and policy-makers must take necessary steps to better understand the nature of diabetes distress, and identify its effects on patients’ health outcomes if they are to improve the overall health of communities. Many researchers believe that diabetes distress and the way it is managed are strong predictors of adopting self-management behaviors in controlling diabetes [11]. This study aims to measure the diabetes distress score and its related factors among patients with type 2 diabetes in Isfahan. The study has the potential to improve our understanding of diabetes-related distress, and can help decision-makers tailor appropriate and well-timed interventions.

## Methods

A cross-sectional study was performed over a period of four months in 2011. Using technique of convenience sampling, a sample of 140 patients was recruited to participate in the study. Patients were eligible to participate if they were over 30 years old with diagnosed diabetes mellitus (type 2) for at least one year, and had attended the relevant training programs about diabetes. The patients were selected on the basis of p ratio between diabetic patients with a confidence level of 95% and 80% power for the test.

Data gathering was performed by calculating Hemoglobin A1c (HbA1c) and a self-reporting scale [12]. HbAlc index was obtained from patients’ medical records. The questionnaire was translated to Persian, with back translation to English. It consisted of two sections including patients’ demographic and health-related information (11 items) and the 17-item Diabetes Distress Scale (DDS-17) which developed by Polonsky et al. in 2005 [13]. The DDS-17 is a self-report scale with four distinct subscales of diabetes-related distress reflecting emotional burden (5 items), physician-related distress (4 items), regimen-related distress (5 items) and interpersonal distress (3 items). The responses to each item were rated on a 6-point frequency scale (1 = not a problem, 2 = a slight problem, 3 = a moderate problem, 4 = somewhat serious problem, 5 = a serious problem and 6 = a very serious problem). According, the minimum and the maximum of the scores in the scale were 17 and 114, respectively. According to Polonsky et al. (2005) a mean item score of three or more (moderate distress) was used as a level of distress worthy of clinical attention. This would help researchers distinguish high from low distress for each item and for the mean of the 17 items (DDS-17). This scale was employed after determining validity and reliability. Content validity method was used to validate the scale; in doing so, the translated scale was given to ten academic staff of Isfahan University of Medical Sciences. Internal reliability of the scale and its four subscales were adequate (α =0.77). Test-retest was used to determine internal reliability of the scale. The first and revised versions of the scale were completed by 30 diabetic patients within two-week interval. The four subscales of DDS scale had Cronbach’s alpha coefficients of 0.81, 0.71, 0.78 and 0.77, respectively.

The results of the pilot study were not included in the main study. SPSS software was used to analyze data, using descriptive tests, chi-square test (χ^2^), ANOVA and Pearson correlation coefficient. Findings were considered to be significant at a conventional level of 0.05. Kolmogorov-Smirnov test was also used to assess the normality of the data prior to data analysis (p > 0.05). Regarding a pathway to better tailor an effective intervention planning for diabetes control, we decided to distinguish which item in each domain has more score. All patients were informed about the purpose of the study and consented to participate in the study. No patient was forced or obliged to participate in the study. This study was approved by Human Research Ethics Committee at Isfahan University of Medical Sciences.

## Results

The response rate was 100%. Patients were aged between 37 and 75 years old with a mean of 53.23 years (SD = 7.82). Almost 54.3% was female, 97.1% was married, and 57.1% had diploma or lower levels of education. The mean duration of diabetes was 7.1 years (SD = 5.63). According to World Health Organization criteria for metabolic control of diabetes, almost 69.3% of patients had borderline metabolism (Table 1).The average score for patients’ diabetes distress was 2.96 ± 0.83; and the average scores for each domain of DDS-17 scale was (3.40 ± 1.18), (2.57 ± 0.88), (2.97 ± 0.90) and (2.76 ± 0.91), respectively. ‘Emotional burden’ was the most significant domain in measuring diabetes-related distress. There was a significant relationship between the total DDS-17 score and patients’ related variables such as age (p = 0.02), duration of living with diabetes (p<0.001), marital status, comorbidity and other complications (p<0.001), as well as history of diabetes (p = 0.01).The relationship between each domain of DDS-17 scale and patients’ socio-demographic and health-related factors have been shown in Table 2.

**Table 1:** Demographic and clinical data

**Variables**	**Frequency (%)**	**Variables**	**Frequency (%)**
Gender		Co morbidity	
Male	64(45.7)	Yes	80(57.1)
Female	76(54.3)	No	60(42.9)
Level of Education		Type of Treatment	
Illiterate	20(14.3)	Oral Agents	92(65.7)
Up to diploma	75(53.6)	Insulin	20(14.3)
Diploma and higher	45(32.1)	Oral Agents & Insulin	28(20)
Marital Status		History of Type2 Diabetes	
Married	136(97.1)	Yes	100(71.4)
Unmarried	4(2.9)	No	40(28.6)
Diabetes Complications		Metabolic control(HbA_1C_)	
Yes	103(73.6)	Optimal control (< 7.0%)	14(10)
No	37(26.4)	Borderline control (7.0- 8.5%) Poor Control (> 8.5%)	97(69.3)29(20.7)

**Table 2:** Relation between mean score of diabetes distress based on sociodemographic and health related variables

**Variables**	**TDD**	**EB**	**PD**	**RD**	**ID**
Gender	NS	NS	NS	NS	NS
Marital Status	<0.001	0.01	<0.001	0.007	NS
Level of Education	NS	0.04	NS	NS	NS
Co morbidity	<0.001	<0.001	0.001	0.002	0.04
Type of Treatment	0.05	NS	NS	NS	NS
History of Type2 Diabetes	0.01	0.001	NS	0.04	NS
Diabetes Complication	<0.001	<0.001	0.002	<0.001	0.007
Metabolic Control(HbA_1C_)	<0.001	<0.001	<0.001	<0.001	<0.001

TDD = Total Diabetes Distress; RD = Regimen distress; PD = Physician Distress

EB = Emotional Burden; ID = Interpersonal distress; NS = Not Significant.

In addition, Pearson correlation coefficient revealed that diabetes distress in patients with type 2 diabetes has a direct relationship with HbA1c (r = 0.63, p<0.001).

## Discussion

The aim of this study was to assess diabetes distress and its related factors in patients with type 2 diabetes in an effort to tailor a good diabetes management intervention. While it has been recognized that diabetes distress is multi-factorial, previous research into diabetes control has attempted to isolate single factors. Identifying and assessing the modifiable determinants of diabetes distress plays a key role in making accurate and appropriate intervention planning programs, to achieve the best possible outcomes [14].

Our findings showed that some patient-related variables including their marital status, having co-morbidity, adherence to medical treatment as well as the type of treatment they receive, the history of type 2 diabetes, their dietary management, having consequent increased risk of diabetes complications and metabolic control have significant correlation with DDS-17 total. In addition, co-morbidity in patients with diabetes mellitus, the consequent occurrence of diabetes complications and metabolic control significantly correlates with all four domains of diabetes distress. These findings are in line with previous research which reported that emotional distress is a strong predictor of diabetic control [15]. Similarly Whittermore et al. showed that social support and self-confidence are the most consistent predictor of metabolic control, dietary self-management, and diabetes-related distress in women living with type 2 diabetes [8].

In this study, we also determined the most rating question for each domain. In emotional burden domain, “feeling angry, scared and/or depressed when I think about living with diabetes” was the most prevalent. It means that our patients had desirable feeling that can affect various dimensions of diabetes care plan, and also their diabetes control. Liu et al. showed that emotional burden is one of the strongest factors of quality of life. For them the assessment of emotional burden is a necessity to make emotional support in diabetes patients, and to improve their quality of life through empowerment strategies [16].

“Feeling that my doctor doesn’t give me clear enough directions on how to manage my diabetes” was the most dominant choice of physician-related distress subscale. Consistent with this finding Lee et al. reported that the mutual trust between patients and their physicians is an important factor in diabetes control as it enhances self-efficacy, adherence, and diabetes outcomes; indicating that the effective interactions between patients and their health professionals can improve the diabetes outcomes [17].

With regards to regimen-related distress, “feeling that I am not testing my blood sugars frequently enough” had the most frequent response within the scale. Patient’s self-monitoring of blood glucose is the key to inspire patients with type 2 diabetes to adopt self-management behaviors [18]. Although patients are cautious about their self-monitoring behaviors, some barriers exist. Allen et al. showed that self-efficacy is an important factor in adherence to self-monitoring behavior [19].

Related to the interpersonal distress subclass was the question of “feeling that friends or family doesn’t give me the emotional support that I would like”. Including patients’ choice points to the perceived social support as a multidimensional aspect in diabetes control. Similar studies have come to the same conclusion. Recent research about diabetes management shows that social support has a beneficial effect on selecting healthy behaviors in their lives including physical activities and nutrition patterns [20-22].

In this study, we also found that diabetes-related distress correlates with HbA1c. The direct relationship between diabetes distress and HbA1c, e.g., means that by increasing diabetes distress score, HbA_1_c is increased and diabetes control becomes worse.

As it was mentioned above, there are several determinants of diabetes-related distress – such as emotional support, the communication between patients and their physicians, self-efficacy and social support – that are essential in diabetes management. Considering these factors, tailoring a patient-centered, collaborative approach to match the fundamental realities of diabetes care becomes a necessity [23]. Patient empowerment approach is widely acknowledged to promote autonomous self-regulation behavior in patients with type 2 diabetes [22]. This approach is mainly designed based on mutual respect and trust, focuses on the value of human life, and is established to ensure equal relationships between patients and healthcare professionals [24].

Due to the important role of diabetes-related distress in improving diabetes control and regime adherence, the assessment of this factor should be integrated into patients’ self-care plan. Arzaghi et al. argue that the Iranian validated Problem Areas in Diabetes scale (IR-PAID-20) plays a critical role in diabetes care for educators [25]. Empowering patients through education necessitates educators to incorporate interactive teaching strategies to better involve patients in problem-solving, and to address their physical, psychological and social needs [24]. Mahjouri et al. developed a valid a reliable scale in attitude for Iranian patients with diabetes which can be changed mine of both patients and educators [26]. Merely providing information often does not lead to diabetes control; patients become more involved in the management of their diabetes if their specific emotional distress are addressed, and have a good sense of social protection, health control, self-efficacy and health beliefs. It is at this interface that healthcare professionals, diabetes educators and policy-makers have the opportunity to foster independence, self-management behaviour and improve patients’ quality of life.

Nonetheless, the study is limited due to selecting rather similar and homogeneous samples and using self-report tool. Future research is advised to focus on developing observational instruments, as well.

## Competing interests

The authors declare that they have no competing interests.

## Authors’ contributions

AT carried out the implementation and data collection of the study, AB participated in the sequence alignment and drafted the manuscript, GSH participated in planning the study and helped to start the study, DSH participated in writing the manuscript, AE participated in analysing statistics of the manuscript, FA participated in planning the study and help in discussion of the manuscript, MMT carried out technical consultation about endocrine matters due to the study which was about type 2 diabetes. All authors read and approved the final manuscript.
